# GC1qR Cleavage by Caspase-1 Drives Aerobic Glycolysis in Tumor Cells

**DOI:** 10.3389/fonc.2020.575854

**Published:** 2020-09-30

**Authors:** Annika Sünderhauf, Annika Raschdorf, Maren Hicken, Heidi Schlichting, Franziska Fetzer, Ann-Kathrin Brethack, Sven Perner, Claudia Kemper, Berhane Ghebrehiwet, Christian Sina, Stefanie Derer

**Affiliations:** ^1^Institute of Nutritional Medicine, University Hospital Schleswig-Holstein, Lübeck, Germany; ^2^Institute of Pathology, University Hospital Schleswig-Holstein, Lübeck, Germany; ^3^Pathology of the Research Center Borstel, Leibniz Lung Center, Borstel, Germany; ^4^Immunology Center, National Heart, Lung, and Blood Institute, National Institutes of Health, Bethesda, MD, United States; ^5^Faculty of Life Sciences and Medicine, School of Immunology and Microbial Sciences, King's College London, London, United Kingdom; ^6^Institute for Systemic Inflammation Research, University of Lübeck, Lübeck, Germany; ^7^Department of Medicine, Stony Brook University, Stony Brook, NY, United States; ^8^1st Department of Medicine, Division of Nutritional Medicine, University Hospital Schleswig-Holstein, Lübeck, Germany

**Keywords:** aerobic glycolysis, gC1qR, inflammasome, mitochondria, OXPHOS, caspase-1, C1qbp, p32/HABP1

## Abstract

Self-sustained cell proliferation constitutes one hallmark of cancer enabled by aerobic glycolysis which is characterized by imbalanced glycolysis and mitochondrial oxidative phosphorylation (OXPHOS) activity, named the Warburg effect. The C1q binding protein (*C1QBP*; gC1qR) is pivotal for mitochondrial protein translation and thus OXPHOS activity. Due to its fundamental role in balancing OXPHOS and glycolysis, *c1qbp*^−/−^ mice display embryonic lethality, while gC1qR is excessively up-regulated in cancer. Although gC1qR encompasses an *N*-terminal mitochondrial leader it is also located in other cellular compartments. Hence, we aimed to investigate mechanisms regulating gC1qR cellular localization and its impact on tumor cell metabolism. We identified two caspase-1 cleavage sites in human gC1qR. GC1qR cleavage by active caspase-1 was unraveled as a cellular mechanism that prevents mitochondrial gC1qR import, thereby enabling aerobic glycolysis and enhanced cell proliferation. *Ex vivo*, tumor grading correlated with non-mitochondrial-located gC1qR as well as with caspase-1 activation in colorectal carcinoma patients. Together, active caspase-1 cleaves gC1qR and boosts aerobic glycolysis in tumor cells.

## Introduction

Proliferation and differentiation of cells comprise cellular processes that require high energy levels. While it is most likely to be a general mechanism that proliferating cells generate their energy via aerobic glycolysis, differentiated post-mitotic cells are known to maintain their energy level via the mitochondrial oxidative phosphorylation (OXPHOS) system (1). Notably, the metabolic switch from cytosolic aerobic glycolysis to the mitochondrial OXPHOS system is suggested to influence the transition of transient amplifying cells into post-mitotic cells (2). However, mechanisms that enable the cells to switch between these metabolic pathways still remain elusive.

Further, it is thought that the metabolic switch from gaining energy primarily via balanced mitochondrial OXPHOS toward aerobic glycolysis, the so-called Warburg effect, is an important driver of tumor formation and proliferation (1, 3–5). Initially, it was hypothesized that tumor cells are characterized mostly by mitochondria dysfunction, while it is now understood that tumor cells still display functional mitochondria (6).

Under chronic inflammation, high cellular proliferation rates are required for proper tissue repair, thereby increasing the possibility of dysregulated cell proliferation and hence inflammation-driven carcinogenesis. Indeed, many tumors and especially colorectal carcinomas (CRCs) develop as a result of a chronic inflammatory microenvironment mediated by pathologically sustained NLRP3 (NACHT, LRR, and PYD domains-containing protein 3) inflammasome activation. This enzymatic complex comprises of NLRP3, ASC (apoptosis-associated speck-like protein containing a CARD) and caspase-1, and is described to be activated by danger-associated molecular patterns including nutrition-derived metabolites such as glucose, fatty acids, cholesterol, ceramide, or uric acid (7) and has been shown to alter metabolic activities of cells by triggering aerobic glycolysis (8). Hence, high-fat or Western diets have been linked to constant NLRP3 inflammasome activation, thereby potentially triggering inflammation-driven carcinogenesis especially in the gut (9, 10).

The mitochondria-located gC1qR [receptor of the globular heads of C1q (11)] protein critically maintains OXPHOS and hence regulates cell metabolism in breast or cervical cancer cells (12–14). Furthermore, studies have shown increased gC1qR expression in most cancer types where it supports metastasis. Thus, augmented gC1qR levels correlate with poor prognosis in cancer patients (15–21). Until now, it is assumed that mitochondrial gC1qR protein maintains mitochondria function by regulating mitochondrial protein translation (22, 23). Of note, while gC1qR is mainly localized to the mitochondria via the presence of an *N*-terminal mitochondrial leader sequence in the protein (22), gC1qR is also present in other subcellular compartments and can be observed on the cell surface of distinct leukocytes (11, 24). However, mechanisms that modulate the mitochondrial localization and thereby metabolic activity of gC1qR in tumor progression still remain to be elusive. Here, we define a novel, caspase-1-mediated, molecular mechanism of controlling gC1qR activity in cells and demonstrate its perturbation in colorectal cancer.

## Materials and Methods

### Study Population

Patients' characteristics are depicted in Table 1. Tissue samples collected from CRC patients utilized in qPCR and Sanger sequencing experiments were purchased from Origene (OriGene Technologies, Inc., Rockville, MD, USA). Colonic biopsy samples collected from CRC patients utilized in retrospective IHC analyses were obtained at the University Hospital Schleswig-Holstein, Campus Lübeck, Germany. Due to the low probability of patients' survival no written informed consents could be obtained retrospectively. The present study was approved by ethical committee of the University of Lübeck (AZ 20-206).

**Table 1:** Overview of study population.

		**Male [*n*]**	**Female [*n*]**	**Median age [y]**	**TNM [*n*]**	**Tumor stage [*n*]**	**Tumor grade [*n*]**	**Tumor cells [%]**
Paired samples from CRC patients for qPCR [*n* = 10]	NormalTumor	5 5	5 5	74.5	n.a.pT3pN0pMX [10]	n.a.IIA [10]	n.a.G1 [3], G2 [7]	n.a. 60–89
Paired samples from CRC patients for IHC analyses [*n* = 5]	Normal Tumor	1 1	4 4	78	n.a. pT2pN0pM0 [1], pT4apN0pM0 [1], pT3pN0pM0 [1], pT4bN0pM0 [1], pT3pN2a M2 [1]	n.a. I [1], IIB [2], IIIB [1], IV [1]	n.a. G1 [2], G2 [1], G3 [1], G4 [1]	n.a.
Tumor samples from CRC patients for qPCR [*n* = 42]	Tumor	19	23	f 72 m 62	pT1pN0pMX [1], pT1pN1pM1 [1], pT2pN0pMX [2], pT2pN1pM1 [1], pT2pN2 MX [3], pT3pN0pM1 [2], pT3pN0pMX [14], pT3pN1pM1 [1], pT3pN1pMX [5], pT3pN2pM1 [2], pT3pN2pMX [4], pT4pN0pMX [2], pT4pN1pMX [3], pT4pN2pMX [1]	I [3], IIA [14]IIB [2], IIIB [8], IIIC [8]IV [7]	G1 [13], G2 [17], G3 [10], G4 [1]	50–95

*CRC, colorectal carcinoma; n, number of patients; n.a., not applicable*.

### Cell Culture

Human colorectal carcinoma cell line HT29-MTX-E12 (Sigma-Aldrich, St. Louis, MO, US) was kept in DMEM medium, the human acute monocytic leukemia cell line THP-1 (Deutsche Sammlung von Mikroorganismen und Zellkulturen, Braunschweig, Germany) was kept in RPMI 1640 medium and the human chronic myelogenous leukemia cell lines HAP1 or HAP1-gC1qR^−/−^ (both from Horizon Discovery, Cambridge, UK) were kept in IMDM medium. All cell culture media were supplemented with 10% (v/v) heat-inactivated FCS, 100 U/ml penicillin, and 100 mg/ml streptomycin. Cells were incubated at 37°C and 5% CO_2_ in a humidified incubator.

THP-1 monocytes were stimulated with 1 μM phorbol 12-myristate 13-acetate (PMA; InvivoGen, San Diego, CA, USA) for 24 h to induce differentiation to THP-1 macrophages. Cells were then pre-incubated with a specific caspase-1 inhibitor (10 μg/ml; Ac-YVAD-cmk from InvivoGen) or the respective control for 60 min. Afterwards, cells were further stimulated with or without lipopolysaccharides (LPS; 100 ng/ml; InvivoGen) for 24 h.

HT29-MTX cells were left untransfected or were transiently transfected with siRNAs (50 μM each) specific for *C1qbp* exon 3 (*C1qbp* siRNA; s2138; Thermo Fisher Scientific, Waltham, MA, USA) or a control siRNA as well as with plasmids encoding human Caspase-1, human NLRP3, and human ASC (all three from InvivoGen, San Diego, CA, USA) or with a mock plasmid by reverse lipofection using Lipofectamine 3000 reagent (Thermo Fisher Scientific) for 96 h. After 24 h of transfection, cells were stimulated for 72 h with 1.25 mM butyrate or were left untreated.

### Generation of HAP1-gC1qR Mutants

The expression plasmid for human wild-type (wt) gC1qR (Sino Biological Inc., Wayne, PA, USA) was utilized for substitution of aspartic acid (D) residues 77 or 229 by glutamic acid (E) (D77E, D229E, or D77E/D229E) using the QuikChange II XL site-directed mutagenesis kit (Agilent, Santa Clara, CA, USA).

HAP1-gC1qR^−/−^ cells were stably transfected with these plasmids, encoding the sequences for gC1qR-wt, gC1qR-D77E, gC1qR-D229E, or gC1qR-D77E/D229E by lipofection using Lipofectamine 3000 reagent (Thermo Fisher Scientific), according to the manufacturer's instructions. Twenty-four hours after transfection, cells were put under selection by adding Hygromycin B (Thermo Fisher Scientific). Stable HAP1-gC1qR mutant cell lines were then further stably transfected with plasmids encoding human Caspase-1, human NLRP3, and human ASC (all three from InvivoGen, San Diego, CA, USA) or with a mock plasmid by lipofection as described above. Selection of successfully transfected cells was performed using Blasticidin (InvivoGen).

### RNA Extraction and Real-Time Quantitative PCR

RNA was extracted using the innuPREP RNA mini kit (Analytik Jena AG, Jena, Germany) and transcribed to cDNA (RevertAid H Minus reverse transcriptase, Thermo Scientific, Schwerte, Germany) using the T Gradient thermocycler (Whatman Biometra, Göttingen, Germany). Real-time quantitative PCR (qPCR) was carried out using Perfecta SYBR Green Supermix, plus specific oligonucleotides using a 96-well-plate format. The amplification program consisted of: (i) preincubation at 95°C for 5 min; (ii) 40 cycles of denaturation at 95°C for 45 s and annealing at appropriate temperature (55°C) for 1 min using the StepOne Plus Real-Time PCR System (ThermoFisher Scientific, Darmstadt, Germany). Melting curve profiles were produced and analyzed following the 2^−dCt^ algorithm. Expression levels were normalized to β*-actin*. The following oligonucleotides were used for analyses (β*-actin*: for: 5′-ACATCCGCAAAGACCTGTACG-3′, rev: 5′-TTGCTGATCCACATCTGCTGG-3′; *C1qbp*: for: 5′-CTGCACACCGACGGAGACAA-3′, rev: 5′-CATATAAGGCCCAGTCCAAG-3′; *Caspase-1*: for: 5′-CAAGACCTCTGACAGCACGT-3′, rev: 5′-GCATCTGCGCTCTACCATCT-3′; *PYCARD*: for: 5′-GAGAACCTGACCGCCGAG-3′, rev: 5′-CCTTCCCGTACAGAGCATCC-3′; *NLRP3*: for: 5′-CGTTCCAGGGAGTCGTTTGA-3′, rev: 5′-GGCCTTCCTTTTCCTCCTCC-3′; *Ldha*: for: 5′-GCACCCAGTTTCCACCATGA-3′, rev: 5′-GCACTCTTCTTCAAACGGGC-3′; *Slc2a1*: for: 5′-TGGCATCAACGCTGTCTTCT-3′, rev: 5′-CTAGCGCGATGGTCATGAGT-3′; *Ki67*: for: 5′-CCTGCTTGTTTGGAAGGG-3′, rev: 5′-CCTGCTTGTTTGGAAGGG-3′; *Fis1*: for: 5′-CAAGGAGGAACAGCGGGATT-3′, rev: 5′-TGCCCACGAGTCCATCTTTC-3′.

### SDS-PAGE and Immunoblotting

Whole-protein extracts were prepared by lysing cells in denaturing lysis buffer containing 1% SDS, 10 mM Tris (pH 7.4), and 1% protease inhibitor mixture (Complete Protease Inhibitor Cocktail; Roche Applied Science, Mannheim, Germany). Protein fractions from the nucleus, the cytosol or the mitochondria/cell membrane were prepared by lysing cells in non-denaturing lysis buffer containing 1% protease inhibitor mixture (Complete Protease Inhibitor Cocktail) and different centrifugation steps. Protein extracts were separated by denaturing SDS-PAGE under reducing conditions and transferred onto polyvinylidene difluoride membranes. After blocking, membranes were probed with primary antibodies specific for human gC1qR (clone 60.11/ab24733 or clone EPR8871/ab131284 both from Abcam, Cambridge, MA, USA; Exon1/3/6 Abs kindly provided by Prof. Berhane Ghebrehiwet), human KLF4 (AF3640, R&D Systems), human TOM20 (#42406), human Caspase-1 (#2225; mainly detects full-length Caspase-1; Supplementary Figure 5E), human NLRP3 (#15101), human ASC (#13833), human pAMPKα (#2535) or AMPKα (#2532), human pAKT (#4060) or AKT (#9272), human HSP60 pp44/42 (#4370) or p44/42 (#4695; all from Cell Signaling Technology, Danvers, MA, US), human VDAC (Sigma-Aldrich, St. Louis, MO), as well as for human HSP60 (#sc-13115; Santa Cruz Biotechnology), washed, and incubated with HRP-conjugated IgG as secondary Ab. Proteins were visualized by chemiluminescence. To determine similar transfer and equal loading, membranes were stripped and reprobed with an Ab specific for β-actin (#4967, Cell Signaling Technology) or for alpha-tubulin (#2125, Cell Signaling Technology).

### ELISA

Supernatants of cell cultures were collected for measurement of IL-1β secretion by specific ELISA (R&D Systems, Inc., Minneapolis, MN, USA) according to the manufacturer's protocol. Release of gC1qR into the cell culture supernatant was determined by diluting the supernatant 1:2 in coating buffer (0.15 g NaH_2_CO_3_, 0.3 g NaHCO_3_, ad 50 ml dH_2_O; pH 9.6). Diluted cell culture supernatants were coated onto a 96-well-microtiter plate over night at 4°C. Next day, gC1qR was detected using a gC1qR-specific primary antibody (anti-Exon3 Ab) in combination with a respective HRP-conjugated secondary antibody. Optical density was measured at 450 nm with a reference wavelength at 540 nm.

### Immunohistochemistry

Immunohistochemical techniques were performed according to standard protocols. Briefly, paraformaldehyde-fixed and de-paraffinized tissue slides were stained with an anti-human gC1qR antibody (clone 60.11 or clone EPR8771; both from Abcam), an anti-human TOM22 antibody (#WH0056993M1, Sigma-Aldrich), an anti-human Caspase-1 antibody (#2225, Cell Signaling Technology) or with respective isotype control antibodies, washed, and incubated with respective HRP-conjugated IgG secondary Abs. Afterwards, tissue slides were incubated with DAB substrate (Dako) and counterstained with Mayer's hemalum solution. In the case of immunofluorescence analyses, slides were incubated with primary antibodies specific for human gC1qR (clone EPR8871; Abcam), HSP60 (#sc-13115; Santa Cruz Biotechnology, Dallas, Texas, USA) or an irrelevant antigen, washed, and incubated with respective fluorochrome-labeled IgG secondary Abs (HSP60: Alexa-Fluor 488 nm; gC1qR: Alexa-Fluor 594 nm; both from ThermoFisher Scientific). Afterwards, slides were counterstained with DAPI (Sigma-Aldrich).

### Caspase-1 Cleavage Assay

**Figure 1:**
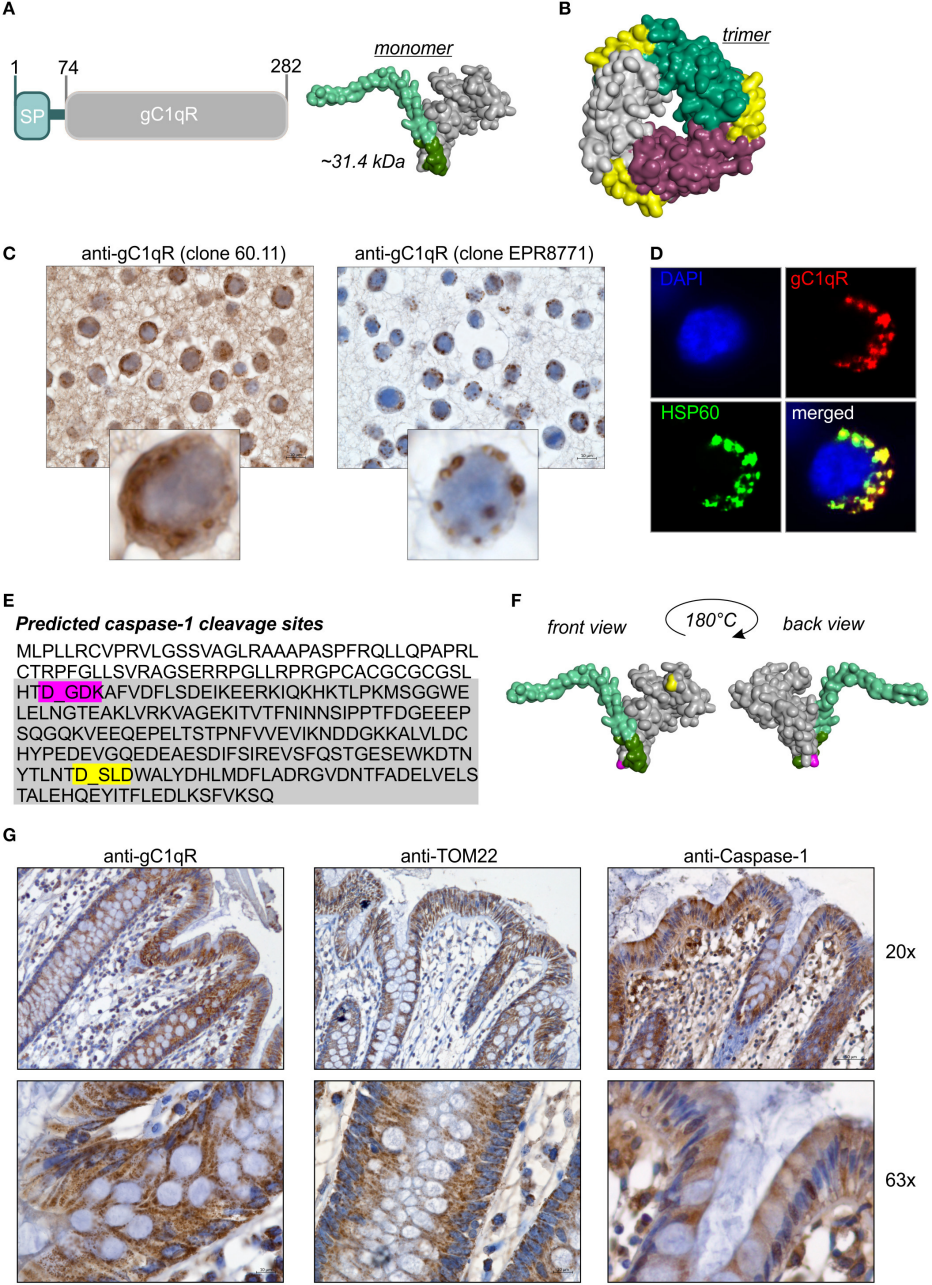
GC1qR displays two caspase-1 cleavage sites. **(A)** Schematic structure model of the human gC1qR protein. SP, signal peptide sequence for mitochondrial import. Homology model of human gC1qR protein was generated using the PHYRE2 server. Bright green = predicted mitochondrial leader sequence, dark green = residual amino acid (aa) residues of exon 1, gray = mature gC1qR. **(B)** Crystal structure of trimeric human gC1qR was generated based on pdb file pdb1p32. **(C)** Immunohistochemistry analyses of paraffin-embedded formalin-fixed HT29 cells using anti-gC1qR Ab clone 60.11 or anti-gC1qR Ab clone EPR8871. **(D)** Co-localization of gC1qR with mitochondrial HSP60 protein in HAP1 cells was assessed by fluorescence microscopy using the anti-gC1qR clone EPR8771. **(E)** *In silico* prediction of potential protease cleavage sites was performed using the PeptideCutter software (https://web.expasy.org/peptide_cutter/). Highlighted in pink = predicted caspase-1 cleavage site at amino acid D77; highlighted in yellow = predicted caspase-1 cleavage site at amino acid D229. **(F)** Predicted caspase-cleavage sites at D77 and D229 were highlighted in pink or yellow, respectively, in the generated homology model of gC1qR. **(G)** Representative pictures from immunohistochemistry analyses of five independent paraffin-embedded formalin-fixed human colonic biopsy samples collected from normal tissue sites from CRC patients using anti-gC1qR Ab (clone EPR8871), anti-TOM22 Ab or anti-Caspase-1 Ab.

Cleavage of human gC1qR by active human caspase-1 was studied by incubating either recombinant human mature His-tagged gC1qR protein (aa 74-282; 30 μg/ml; Supplementary Figure 1F; Prospec, East Brunswick NJ, USA), recombinant human full-length GST-tagged gC1qR protein (aa 1-282; 30 μg/ml; Figures 1D–G; Abnova, Walnut, CA, USA) or native protein lysates isolated from HAP1 cells in the presence or absence of human active caspase-1 (600 U/ml; Enzo Life Sciences GmbH, Lörrach, Germany) for 4 h at 37°C in a water bath. The reaction was stopped by the addition of a reducing SDS-buffer and heating at 95°C for 5 min. Reaction samples were then separated by denaturing SDS-PAGE and proteins were either transferred onto polyvinylidene difluoride membranes for immunoblot analyses or were stained with Coomassie blue. Protein bands were cut out of the Coomassie stained gel. Protein spots were in-gel digested by trypsin and analyzed by nanoHPLC-ESI-MS/MS method at the company Proteome Factory AG (Berlin, Germany).

### Analysis of Cell Proliferation

The CellTiter 96® AQueous Non-Radioactive Cell Proliferation Assay (MTS) that measures metabolic activity of cells was performed using parental HAP1 cells or HAP1-gC1qR^−/−^ cells (5 × 10^3^ cells per well in a 96-well-microtiter plate, 72 h incubation at 37°C and 5% CO_2_) according to the manufacturer's instructions (Promega, Madison, WI, USA).

The neutral-red cytotoxicity assay was performed to determine viable cell mass in HAP1-gC1qR wt or mutant cell lines. 5 × 10^3^ cells per well were seeded into a 96-well-microtiter plate and incubated for 96 h at 37°C and 5% CO_2_. After incubation, cells were stained using a neutral red dye (Sigma-Aldrich), washed and destained to release incorporated dye into the supernatant. Neutral-red dye uptake of analyzed cells was then analyzed by measuring the absorbance at 540 and 690 nm in a microplate reader.

### Seahorse XF Cell Mito Stress Test

The Seahorse XF24 Cell Mito Stress Test was performed with parental HAP1 cells (3 × 10^4^ cells/well), HAP1-gC1qR^−/−^ cells (6 × 10^4^ cells/well), HAP1-mock-NAC (4 × 10^4^ cells/well), HAP1-gC1qR-wt-NAC (2 × 10^4^ cells/well), or HAP1-gC1qR-D77E/D229E-NAC (2 × 10^4^ cells/well), 5 μM FCCP, 10 μM oligomycin, and 5 μM rotenone/antimycin A according to the manufacturer's instructions (Agilent). In the case of HAP1-gC1qR^−/−^ and parental HAP1 cells, cells were seeded 24 h before running the Seahorse XF24 Cell Mito Stress Test. In the case of HAP1-mock-NAC, HAP1-gC1qR wt-NAC, and HAP1-gC1qR D77E/D229E-NAC transfectants, cells were seeded 48 h before running the Seahorse XF24 Cell Mito Stress Test. Cells were counted at the end of the assay and OCR and ECAR were normalized to cell count. In the case of HT29-MTX cells, 5 × 10^3^ cells/well were seeded in 5 mM glucose containing DMEM medium and were left untreated or stimulated with 1.25 mM butyrate for 24 h. Basal OCR and ECAR were measured in standard Seahorse medium according to the manufacturer's instructions (Agilent).

### Extracellular Oxygen Consumption Assay

The consumption of extracellular oxygen to drive oxidative phosphorylation was determined in HAP1-gC1qR wt or mutant cell lines (1 × 10^5^ cells per well in a 96-well-microtiter plate) using the Extracellular Oxygen Consumption Assay according to the manufacturer's instructions (Abcam).

### Determination of Lactate Production

The L-lactic acid assay was performed using supernatants (diluted 1:20 in 1 × PBS in the case of HAP1 cells or diluted 1:10 in 1 × PBS in the case of THP-1 cells) collected from indicated cell lines according to the manufacturer's instructions (Megazyme, Co Wicklow, Ireland). Data were normalized to the cell count in the case of THP-1 cells as well as to values received from respective neutral-red cytotoxicity assays in the case of HAP1 cells.

### Statistical Analysis

Data are displayed graphically and were statistically analyzed using GraphPad Prism 6.0. Curves were fitted using a non-linear regression model with a sigmoidal dose response (variable slope if applicable). Statistical significance was determined by the one-way or two-way ANOVA repeated measures test with the Bonferroni posttest. If not stated otherwise, the respective results were displayed as mean ± SEM of at least three independent experiments. The *p*-values were calculated and null hypotheses were rejected when *p* ≤ 0.05.

## Results

### GC1qR Comprises Two Potential Caspase-1 Cleavage Sites

The full-length human gC1qR protein (282 amino acids; 31.4 kDa) includes an *N*-terminal signal leader sequence for mitochondrial import (Figure 1A) and forms trimeric complexes (Figure 1B) (11, 20, 25). Although the gC1qR activity on mitochondria is best studied, the protein is also found in the cytosol, the cell membrane and the extracellular compartment but its functional activity at these locations is unclear (24, 26). In the present study, distinct cellular localizations in the human colorectal carcinoma cell line HT29-MTX were visualized by immunohistochemistry (IHC) experiments utilizing gC1qR antibodies recognizing either the *N*-terminal residues 76-93 (clone 60.11) or the *C*-terminal residues 213-226 (clone EPR8871). As depicted in Figure 1C, the antibody clone 60.11 detected cytosolic and mitochondrial gC1qR, while the antibody clone EPR8871 mainly detected mitochondrial gC1qR in HT29-MTX cells (Figure 1C), indicating that gC1qR is present in distinct protein forms in these cells. Detection of mitochondrial gC1qR was further validated by co-localization experiments showing that fluorescence signals from the anti-gC1qR antibody (clone EPR8871) overlaid with fluorescence signals from an anti-HSP60 antibody, a protein mainly localized to mitochondria (Figure 1D).

To unravel mechanisms regulating localization of gC1qR to distinct cellular compartments, we first hypothesized that the *N*-terminal mitochondrial leader (Figure 1A) may be removed to prevent mitochondrial localization and promote localization to other cellular compartments. Hence, we performed an *in silico* prediction analysis of potential protease cleavage sites in human gC1qR protein using the PeptideCutter server (https://web.expasy.org/peptide_cutter/). Unexpectedly, caspase-1 was predicted to cleave at two distinct sites [amino acid (aa) residues 77 and 229] in the gC1qR protein sequence besides conventional protease cleavage sites. The first consensus sequence for caspase-1 cleavage was identified between aa 77-80 (DGDK) and the second one between aa 229-232 (DSLD) (Figure 1E). Notably, the first caspase-1 cleavage site in gC1qR is located directly after exon 1 (aa 1-74) that encodes the mitochondrial leader (Figure 1F). Next, we examined colonic expression of gC1qR, of the mitochondrial protein TOM22 as well as of caspase-1 by IHC experiments utilizing human normal biopsy samples. Indeed, high expression level of gC1qR, TOM22 as well as of caspase-1 were detected in colonic intestinal epithelial cells (IECs) as well as in lamina propria leukocytes, indicating a potential interaction between gC1qR and caspase-1 in these cells (Figure 1G, Supplementary Figure 1).

### Caspase-1 Cleaves gC1qR at Amino Acid Residues 77 and 229

**Figure 2:**
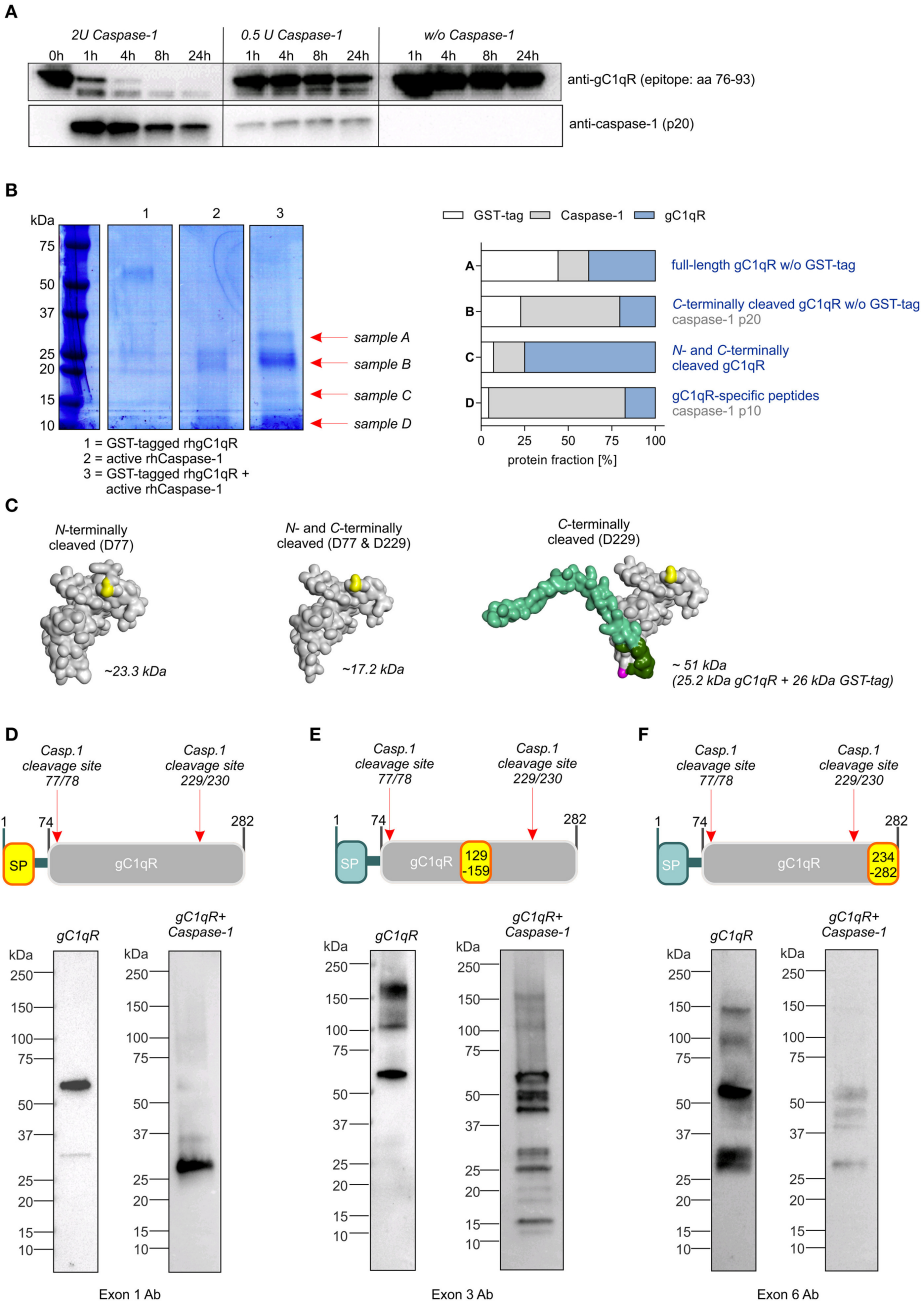
Caspase-1 cleaves gC1qR at amino acid residues 77 and 229. **(A)** *In vitro* cleavage assay was performed by incubating human recombinant gC1qR (~32 kDa) in the presence or absence of human recombinant active caspase-1 (~10 and 20 kDa) at 37°C and indicated time periods. Afterwards, reduced protein samples were separated by SDS-PAGE. Western blot experiments were performed using an anti-gC1qR antibody (clone 60.11) or an anti-caspase-1 antibody. **(B)** *In vitro* cleavage assay was performed by incubating *N*-terminally GST-tagged human recombinant gC1qR (~58 kDa) in the presence or absence of human recombinant active caspase-1 (~10 and 20 kDa) at 37°C for 24 h. Afterwards, reduced protein samples were separated by SDS-PAGE and proteins were visualized by coomassie blue staining. From lane 3 indicated protein bands were cut out of the gel. Protein spots were in-gel digested by trypsin and analyzed by nanoHPLC-ESI-MS/MS method. Fractions of peptides specific for cleaved or non-cleaved human gC1qR, for active caspase-1 or for GST in analyzed protein samples (A–D) are presented in the right panel. **(C)** Homology models of *N*-terminally cleaved, N-and C-terminally cleaved or C-terminally cleaved human gC1qR protein were generated using the PHYRE2 server. Full-length gC1qR has a calculated molar mass of 31.4 kDa, while *N*-terminally cleaved gC1qR has a calculated mass of 23.3 kDa. C-terminally cleaved gC1qR has a molar mass of 25.2 kDa (or in its GST-tagged form 51.2 kDa) and double-cleaved gC1qR has a molar mass of 17.2 kDa. **(D–F)** Western blot experiments using distinct gC1qR-directed primary antibodies with different binding epitopes located in exon 1, exon 3, or exon 6.

To verify predicted caspase-1 cleavage sites in human gC1qR protein, an *in vitro* cleavage assay was performed using recombinant human gC1qR protein (rhgC1qR) in combination with different concentrations and incubation times of recombinant human active caspase-1. As presented in Figure 2A, Western blot experiments against gC1qR revealed gC1qR to be cleaved in a time- and concentration-dependent manner by active caspase-1 (Figure 2A). Additionally, an *in vitro* cleavage assay was performed utilizing *N*-terminally GST-tagged rhgC1qR that also included fractions of untagged full-length rhgC1qR (Supplementary Figures 2A,B). Samples derived from this *in vitro* cleavage assay that contained either rhgC1qR or active recombinant human caspase-1 alone as well as rhgC1qR combined with active rhcaspase-1 were separated by SDS-PAGE and stained with Coomassie blue. Afterwards, four specific protein bands (indicated as sample A-D) were isolated and peptide sequencing was performed that validated predicted caspase-1 cleavage sites in human gC1qR protein at amino acid residues 77 and 229 (Figure 2B, Supplementary Figure 2C). Cleavage of gC1qR by active caspase-1 was further verified by Western blot experiments utilizing primary antibodies specific for exon 1 (aa1-74), exon 3 (aa129-159) and exon 6 (aa234-282). In the presence of active caspase-1, anti-exon1-Ab only detected *C*-terminally cleaved gC1qR w/o GST-tag (~25.2 kDa; Figures 2C,D), anti-Exon3-Ab detected various cleavage products including *N*-and *C*-terminally cleaved fragments (*N*-terminally cleaved gC1qR ~23.3 kDa; C-terminally cleaved gC1qR ~25.2 kDa or 51 kDa; *N*- and *C*-terminally cleaved gC1qR ~17.2 kDa; Figures 2C,E) and anti-Exon6-Ab displayed a mere loss of binding, potentially indicating caspase-1 to first cut at aa229 (Figure 2F).

### Deficiency of gC1qR Expression Induces Loss of OXPHOS Activity

**Figure 3:**
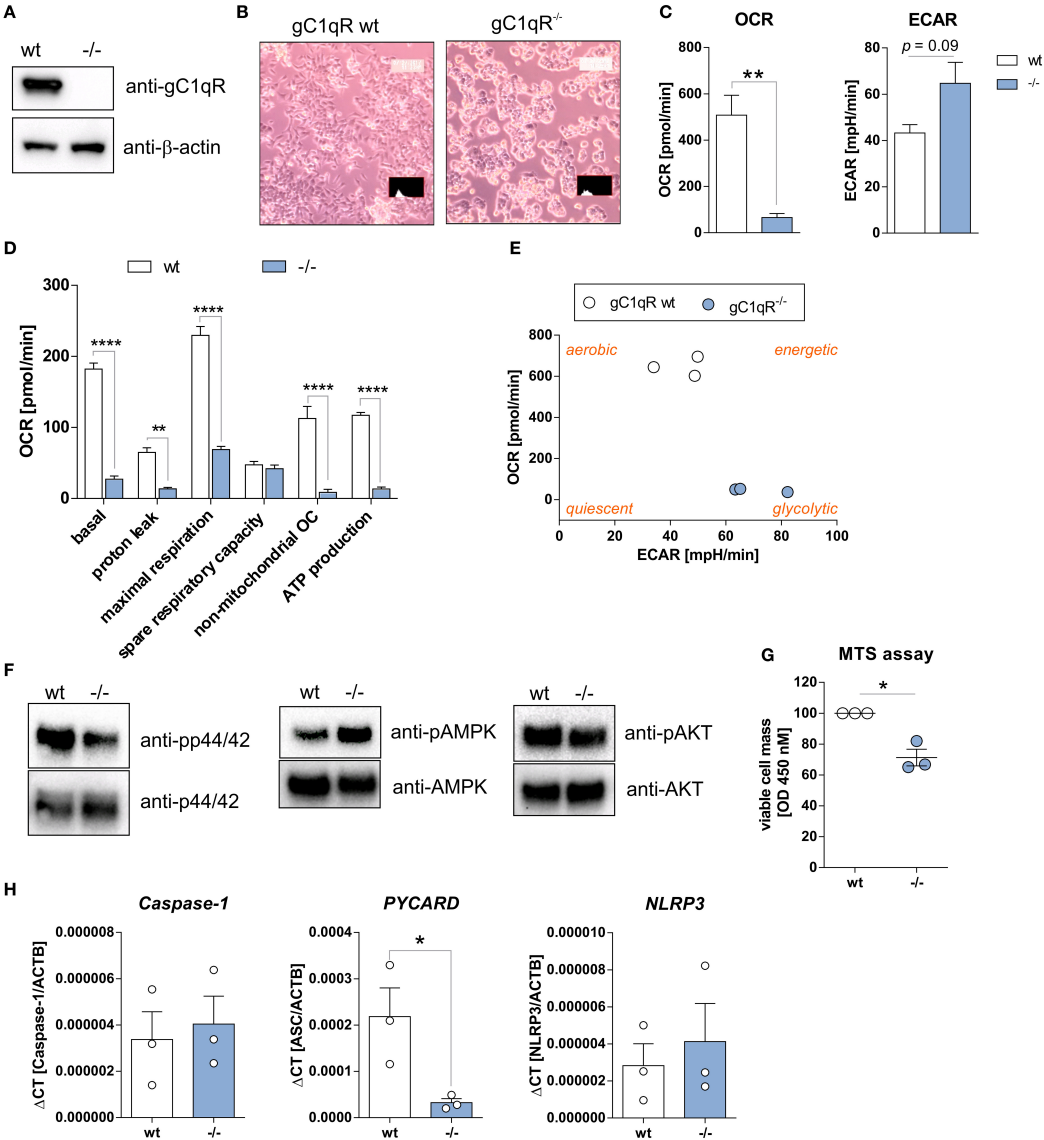
HAP1-gC1qR knockout cells display loss of OXPHOS and cell proliferation activity. **(A)** Whole protein extracts were isolated from parental HAP1 cells that express wild-type gC1qR (wt) and HAP1-gC1qR knockout cells (–/–). Western blot analyses were performed using primary antibodies specific for gC1qR (60.11 Ab) or β-actin. **(B)** Cellular morphology of parental HAP1 cells and HAP1-gC1qR knockout cells was assessed by light microscopy. **(C,D)** Oxygen consumption rate (OCR) of HAP1-gC1qR-wt and HAP1-gC1qR^−/−^ cells was determined using the Seahorse XF Cell Mito Stress Test. **(E)** Graphical presentation of data received from the Seahorse XF Cell Mito Stress Test to visualize cellular metabolic state. **(F)** Whole protein extracts were isolated from parental HAP1 and ^−^/^−^ cells. Western blot analyses were performed using primary antibodies specific for pp44/42, p44/42, pAMPKα, AMPKα, pAKT, AKT or β-actin. **(G)** Wt and –/– cells were cultivated for 72 h and cell viability was assayed by MTS assay. **(H)** The mRNA expression level of *Caspase-1, PYCARD*, and *NLRP3* in wt and –/– cells were quantified by qPCR. Results are expressed as mean ± SEM and are presented from at least three independent experiments. *p ≤ 0.05, **p ≤ 0.01, ****p ≤ 0.0001.

To study functional consequences of the cleavage of gC1qR by active caspase-1 we utilized the human haploid HAP1 cell line system in which gC1qR was knocked-out by CRISPR/Cas9 technology. As demonstrated by Western blot experiments, we verified the loss of gC1qR protein in the HAP1-gC1qR knock-out cells (–/–) in comparison to the parental HAP1 cell line that expresses wild-type gC1qR (Figure 3A). Furthermore, HAP1-gC1qR^−/−^ cells displayed a distinct cellular morphology (Figure 3B), a complete loss of basal and maximal OXPHOS activity as well as of non-mitochondrial respiration but similar spare respiratory capacity and enhanced extracellular acidification rate (ECAR) in comparison to the parental cell line as determined by Seahorse XF Cell Mito Stress test (Figures 3C,D).

Overall decrease in oxygen consumption together with significantly decreased ATP levels indicated low metabolic activity of HAP1-gC1qR^−/−^ cells. Hence, while a highly balanced OXPHOS activity was detected in the parental HAP1 cell line, the HAP1-gC1qR^−/−^ cells were found to preferentially perform anaerobic glycolysis (Figure 3E). These differences in cellular metabolism were reflected by decreased p44/42 and AKT but enhanced AMPK activation (Figure 3F), resulting in significantly diminished cell viability in HAP1-gC1qR^−/−^ cells (Figure 3G). However, parental HAP1 cells as well as HAP1-gC1qR^−/−^ cells were found to express extremely low mRNA level of the inflammasome components *Caspase-1, PYCARD*, or *NLRP3* (Figure 3H) that are fundamental for efficient caspase-1 activation.

### Cleavage of gC1qR by Active Caspase-1 Promotes Aerobic Glycolysis

**Figure 4:**
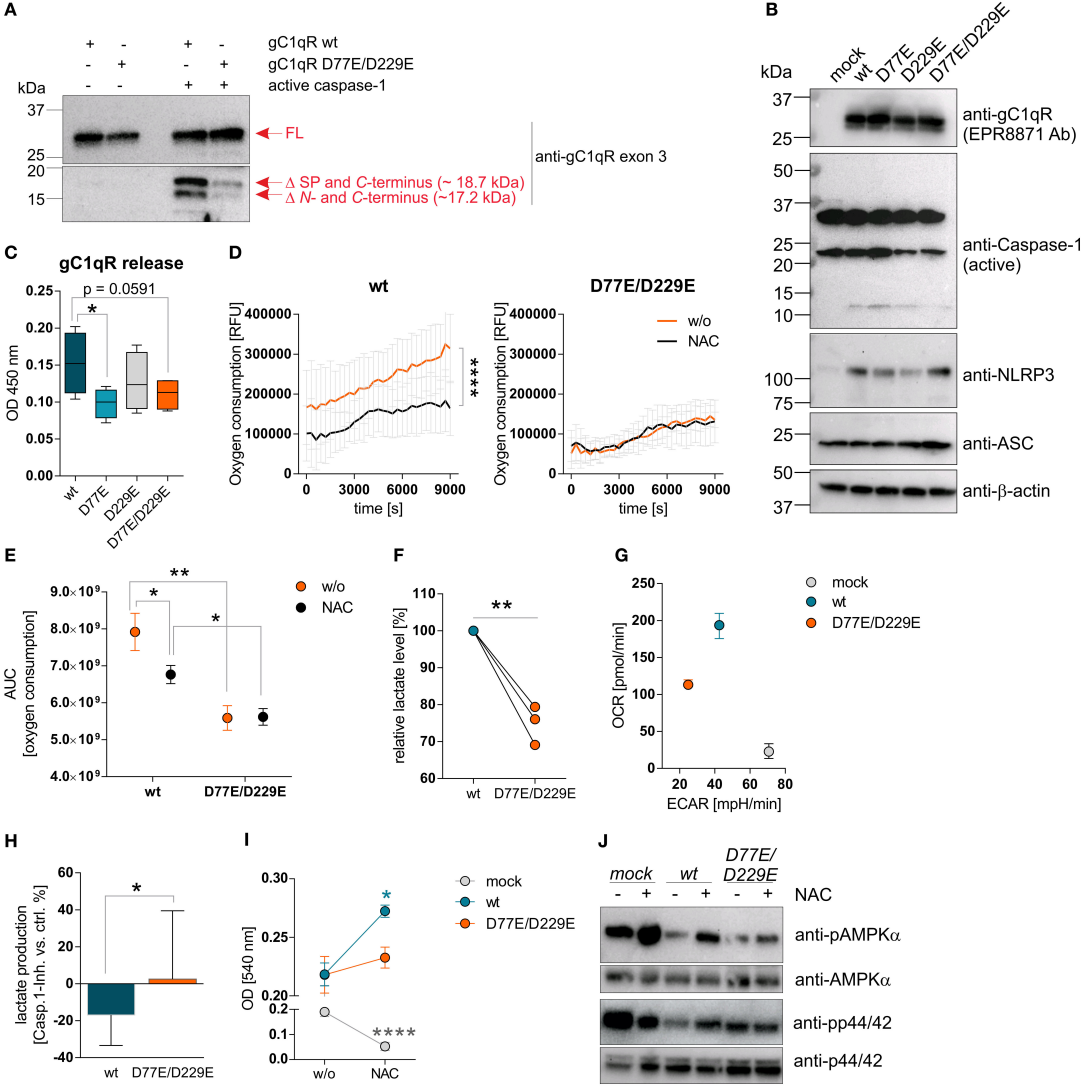
Cleavage of gC1qR by active caspase-1 results in decreased OXPHOS activity and enhanced proliferation. **(A)** Site-directed mutagenesis of human *C1qbp* was performed using site-specific oligonucleotides to prevent caspase-1 cleavage of gC1qR. Aspartic acid **(D)** residues at aa77 and aa229 were substituted by glutamic acid **(E)** residues, resulting in the following gC1qR mutant D77E/D229E. HAP1-gC1qR^−/−^ cells were stably transfected with generated plasmids encoding wt or mutated *C1qbp* variant D77E/D229E. *In vitro* cleavage assay was performed by incubating whole native protein lysates extracted from HAP1-gC1qR-wt or HAP1-gC1qR-D77E/D229E cells in the presence or absence of human recombinant active caspase-1 at 37°C for 4 h. Afterwards, reduced protein samples were separated by SDS-PAGE. Western blot experiments were performed using a primary antibody specific for exon 3 of human gC1qR. SP, signal peptide for mitochondrial import; Δ, cleavage; FL, full-length. **(B)** Plasmids encoding the human inflammasome components caspase-1 **(C)**, NLRP3 (N) or ASC (A) were stably transfected into generated HAP1-gC1qR wt or mutant cell lines. Whole protein extracts were isolated and western blot analyses were performed using primary antibodies specific for gC1qR (clone EPR8871), caspase-1, NLRP3, ASC, or b-actin. **(C)** GC1qR release was measured by gC1qR-specific ELISA (exon 3 Ab) in supernatants from HAP1-gC1qR transfectants stably expressing all three inflammasome components NAC after 72 h of incubation. **(D)** Time-dependent measurement of oxygen consumption rate of indicated HAP1 transfectants in the presence or absence of over-expressed inflammasome components (NAC). **(E)** The area under the curve of data presented in **(D)** was calculated for each single experiment and each cell line. **(F)** Lactate production was measured in cell culture supernatants after 72 h of incubation of HAP1-gC1qR wt or D77E/D229E mutant cell lines stably transfected with NAC. Lactate levels generated by gC1qR-D77E/D229E mutant were related to lactate level produced by gC1qR-wt cells. **(G)** Oxygen consumption rate (OCR) as well as extracellular acidification rate (ECAR) of HAP1-gC1qR-wt-NAC, HAP1-gC1qR-D77E/D229E-NAC as well as HAP1-mock-NAC cells were determined using the Seahorse XF Cell Mito Stress Test. Results from triplicates from one representative experiment are presented. **(H)** Lactate level were determined in cell culture supernatants collected from HAP1-gC1qR-wt-NAC or HAP1-gC1qR-D77E/D229E-NAC cells that have been incubated in the absence or presence of 100 μg/ml caspase-1 inhibitor for 72 h. Results received from caspase-1 inhibitor treated cells were related to results from control treated cells for each cell line. **(I)** Cell viability of NAC expressing or NAC non-expressing HAP1-gC1qR-wt, HAP1-gC1qR-D77E/D229E, or HAP1-mock cells was determined after 72 h incubation by the neutral red assay. Optical density was measured at 540 nm. **(J)** Whole protein extracts were isolated from NAC expressing or NAC non-expressing HAP1-gC1qR-wt, HAP1-gC1qR-D77E/D229E, or HAP1-mock cells. Western blot analyses were performed using primary antibodies specific for pAMPKα, AMPKα, pp44/42, or p44/42. Results are expressed as mean ± SEM and are presented from at least three independent experiments. *p ≤ 0.05, **p ≤ 0.01, ****p ≤ 0.0001.

To investigate functional consequences of gC1qR cleavage at aspartic acid residues 77 (D77) or 229 (D229) by active caspase-1, we established distinct HAP1-based cell lines stably expressing either a mock plasmid, wild-type gC1qR (wt), mutant gC1qR-D77E, mutant gC1qR-D229E, or mutant gC1qR-D77E/D229E. Of note, gC1qR protein was localized to the cellular organelle protein fraction, including the mitochondria protein fraction, but not to the cytosol or to the nucleus in all established HAP1-gC1qR mutant cell lines (Supplementary Figures 3A–C). As depicted in Figure 4, substitution of aspartic acid residues 77 and 229 by glutamic acid residues prevented active caspase-1 mediated cleavage of gC1qR in HAP1 cells in an *in vitro* cleavage assay utilizing recombinant human active caspase-1 in combination with native protein lysates isolated from indicated HAP1 cells (Figure 4A). Furthermore, generated HAP1 transfectants were additionally stably transfected with plasmids encoding human caspase-1 (C), human ASC (A) and human NLRP3 (N), all three plasmids (NAC), or were left untransfected (w/o NAC) (Figure 4B). Constant overexpression of all three inflammasome components resulted in the release of gC1qR into the cell culture supernatant in gC1qR-wt cells that was significantly decreased in gC1qR-D77E and gC1qR-D77E/D229E but not in gC1qR-D229E mutant cell lines (Figure 4C). This suggests that cleavage of gC1qR by caspase-1 at amino acid residue D77 prevents mitochondria import and triggers release of gC1qR to the extracellular compartment.

Next, we studied extracellular oxygen consumption by these HAP1 transfectants. We detected highest oxygen consumption in HAP1-gC1qR-wt w/o NAC cells (RFU = 314,140 ± 85,329) in comparison to HAP1-gC1qR-D77E w/o NAC cells (RFU = 258,942 ± 84,888), to HAP1-gC1qR-D229E w/o NAC cells (RFU = 156,765 ± 55,276) or to HAP1-gC1qR-D77E/D229E w/o NAC cells (RFU = 135,065 ± 19,981), revealing aspartic acid residues 77 and 229 to be crucial for efficient mitochondrial respiratory capacity. Due to the findings that HAP1 cells endogenously express low level of the NLRP3 inflammasome components (Figure 3H), we cannot exclude low basal caspase-1 activity that may alter mitochondrial import of functional gC1qR protein. Hence, one may hypothesized that these stable transfectants have been adapted to metabolic changes, therefore potentially displaying lower OXPHOS activity, as mainly observed for the gC1qR-D229- and gC1qR-D77E/D229E-mutants, in comparison to gC1qR-wt cells. Of note, overexpression of all three inflammasome components NAC resulted in a significant loss of oxygen consumption in HAP1-gC1qR-wt or HAP1-gC1qR-D77E mutants (Figures 4D,E, Supplementary Figures 4A,B). Furthermore, lactate production was highest in gC1qR-wt-NAC cells, followed by gC1qR-D77E-NAC cells (fold change = −1.1) and was significantly reduced in gC1qR-D229-NAC (fold change = −1.5) and gC1qR-D77E/D229E-NAC cells (fold change = −1.3) compared to gC1qR-wt-NAC cells (Figure 4F, Supplementary Figure 4C). Hence, we hypothesized that gC1qR cleavage by active caspase-1 results in the loss of OXPHOS activity thereby inducing an imbalance between glycolysis and OXPHOS, potentially enabling increased activity of the pentose-phosphate pathway (1). These findings were further verified by the Seahorse XF Cell Mito Stress test that identified HAP1-gC1qR-wt NAC cells to perform aerobic glycolysis, while the double-mutant HAP1-gC1qR-D77E/D229E NAC cells displayed a loss of OXPHOS as well as of the ECAR, indicating that these cells were metabolically less active. Again, high anaerobic glycolysis activity was detected in mock-NAC transfected HAP1 cells (Figure 4G). Of note, higher lactate production in HAP1-gC1qR-wt NAC cells in comparison to double-mutant HAP1-gC1qR-D77E/D229E NAC cells (Figure 4F) was diminished by pharmacological inhibition of caspase-1 by about ~17% (Figure 4H). Additionally, overexpression of the inflammasome components NAC were identified to significantly boost cell proliferation in gC1qR-wt but not in gC1qR-D77E/D229E cells, pointing to a critical role of gC1qR cleavage by active caspase-1 in the induction of cell proliferation. On the other side, mock transfected HAP1 cells died in the presence of active caspase-1, further underlining the findings that gC1qR seems to be indispensable for maintaining OXPHOS activity for efficient energy supply under stress conditions (Figure 4I). These data were reflected by phosphorylation states of AMPKα and p44/42 (Figure 4J). Of note, if caspase-1 mediated cleavage of gC1qR cannot occur due to the knockout of respective cleavage site, cell proliferation should be reduced and the cells will shift to a more quiescent cell state, reflected by low OXPHOS and glycolysis activities. Indeed, this is what we observe for the single D229E-mutant and for the double-mutant D77E/D229E cells. Hence, we hypothesize that *C*-terminal cleavage of gC1qR by caspase-1 between aa 229 an 230 may be most critical for shifting the cells into a proliferative and metabolically active state.

**Figure 5:**
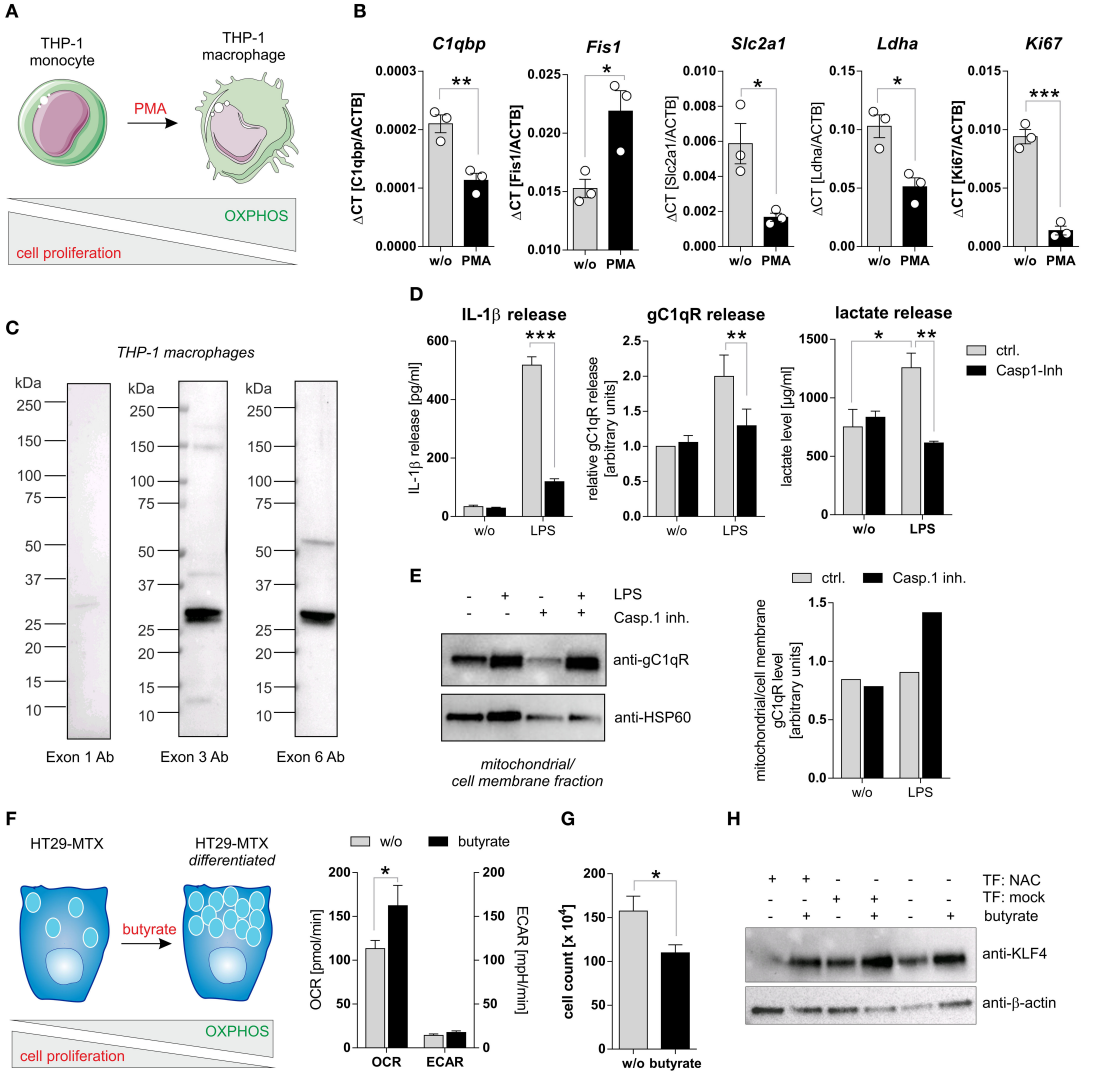
Caspase-1 activation enhances glycolysis and impairs cell differentiation. **(A)** Schematic model of PMA induced differentiation of THP-1 monocytes into macrophages. This figure was generated using pictures provided by the Servier Medical Art homepage https://smart.servier.com/. Servier Medical Art by Servier is licensed under a Creative Commons Attribution 3.0 Unported License. **(B)** QPCR analyses were performed to quantify mRNA expression level of *C1qbp, Fis1, Slc2a1, Ldha*, and *Ki67*. **(C)** Western blot analysis of whole protein extracts from PMA-induced differentiated THP-1 macrophages using gC1qR-directed antibodies specific for epitopes located in exon 1, 3, or 6. **(D)** Secretion of IL-1β (left panel), gC1qR (middle panel) or lactate (right panel) was determined using specific ELISA using supernatants from untreated or LPS stimulated PMA-differentiated THP-1 cells in the presence or absence of caspase-1 inhibitor (10 μg/ml; Ac-YVAD-cmk from InvivoGen). **(E)** GC1qR protein expression in mitochondrial/ cell membrane protein fractions was quantified by Western blot experiments. PMA-differentiated THP-1 cells were stimulated with LPS in the presence or absence of caspase-1 inhibitor (10 μg/ml; Ac-YVAD-cmk) or were left untreated. Densitometry was performed using the software ImageJ (right panel). **(F)** Schematic model of butyrate induced goblet cell differentiation of HT29-MTX cells (left panel). Oxygen consumption rate (OCR) as well as extracellular acidification rate (ECAR) of HT29-MTX cells stimulated with 1.25 mM butyrate for 24 h or left untreated were determined using the Seahorse XF Cell Mito Stress Test (right panel). **(G)** HT29-MTX cells were incubated in the absence or presence of 1.25 mM butyrate for 72 h. After incubation, cells were counted. **(H)** HT29-MTX cells were transiently transfected for 96 h with plasmids encoding full-length human caspase-1 (C), human ASC (A), human NLRP3 (N) or with a mock plasmid in the presence or absence of 1.25 mM butyrate. Whole protein extracts were separated by SDS-PAGE under reducing conditions and Western blot experiments were performed using indicated primary antibodies. Results are presented as mean ± SEM from at least three independent experiments. *p ≤ 0.05, **p ≤ 0.01, ***p ≤ 0.001.

To study caspase-1 mediated cleavage of gC1qR in a second cell system that endogenously expresses wild-type gC1qR and all three inflammasome components (NAC) we utilized PMA-induced differentiated THP-1 macrophages (Figure 5A). Differentiated THP-1 macrophages in comparison to THP-1 monocytes were demonstrated to display significantly decreased mRNA expression levels of *C1qbp*, of the glucose transporter *Slc2a1*, of the lactate dehydrogenase a (*Ldha*) as well as of the proliferation marker *Ki67*. Of note, the mitochondria fission protein 1 (Fis1) was significantly upregulated in THP-1 macrophages (Figure 5B). Hence, PMA-induced differentiation of THP-1 monocytes into macrophages initiates metabolic reprogramming of THP-1 cells, characterized by diminished metabolic activity and reduced cell proliferation. Notably, low full-length gC1qR protein expression (anti-exon 1 Ab) but strong mature gC1qR protein expression (anti-exon 3 Ab and anti-exon 6 Ab) was detected in THP-1 macrophages by Western blot experiments (Figure 5C). These findings point to the loss of the mitochondria leader, located in exon 1, possibly due to mitochondria import but not to cleavage of gC1qR by active caspase-1 due to the presence of exon 6. Furthermore, LPS stimulation of THP-1 macrophages resulted in caspase-1 mediated induction of glycolysis, reflected by significantly increased secretion of IL-1β (Figure 5D, left panel), gC1qR (Figure 5D, middle panel) as well as of lactate (Figure 5D, right panel) that all were significantly blocked by pharmacological caspase-1 inhibition. The finding that active caspase-1 prevents gC1qR mitochondria localization being associated with enhanced glycolysis was supported by Western blot experiments demonstrating increased gC1qR localization to the mitochondrial/cell membrane protein fraction in LPS stimulated THP-1 macrophages in the presence of a caspase-1 inhibitor (Figure 5E).

### Activation of Caspase-1 Impairs Differentiation of Colorectal Carcinoma Cells

To strengthen the hypothesis that caspase-1 mediated cleavage of gC1qR protein prevents its localization to mitochondria and hence critically impacts cellular metabolism, we additionally utilized the colorectal carcinoma cell line HT29-MTX in functional analyses. Stimulation of HT29-MTX cells with the OXPHOS promoting short chain fatty acid (SCFA) butyrate has been previously demonstrated by our group and other groups to enhance differentiation of these cells into mucus-producing goblet cells [Figures 5F–H; unpublished data from our laboratory; (27, 28)]. Indeed, HT29-MTX cells stimulated with butyrate displayed a significantly enhanced OXPHOS activity, being reflected by an increased oxygen consumption rate, an unaltered extracellular acidification rate (Figure 5F) and significantly decreased cell proliferation (Figure 5G). To study the effect of caspase-1 activation on butyrate induced differentiation of HT29-MTX cells, cells were transiently transfected with plasmids encoding all three inflammasome components (NAC) before stimulation with butyrate. Of note, constant and butyrate-induced goblet cell differentiation was decreased in the presence of active NLRP3 inflammasome, indicated by reduced expression of the goblet cell marker KLF4 [(29); Figure 5H]. Together, these findings further highlight the critical role of active caspase-1 in mediating metabolic reprogramming of tumor cells, leading to the loss of tumor cells‘ differentiation state.

### Non-mitochondrial gC1qR Protein Expression Correlates With Grading of Colorectal Carcinoma Cells

**Figure 6:**
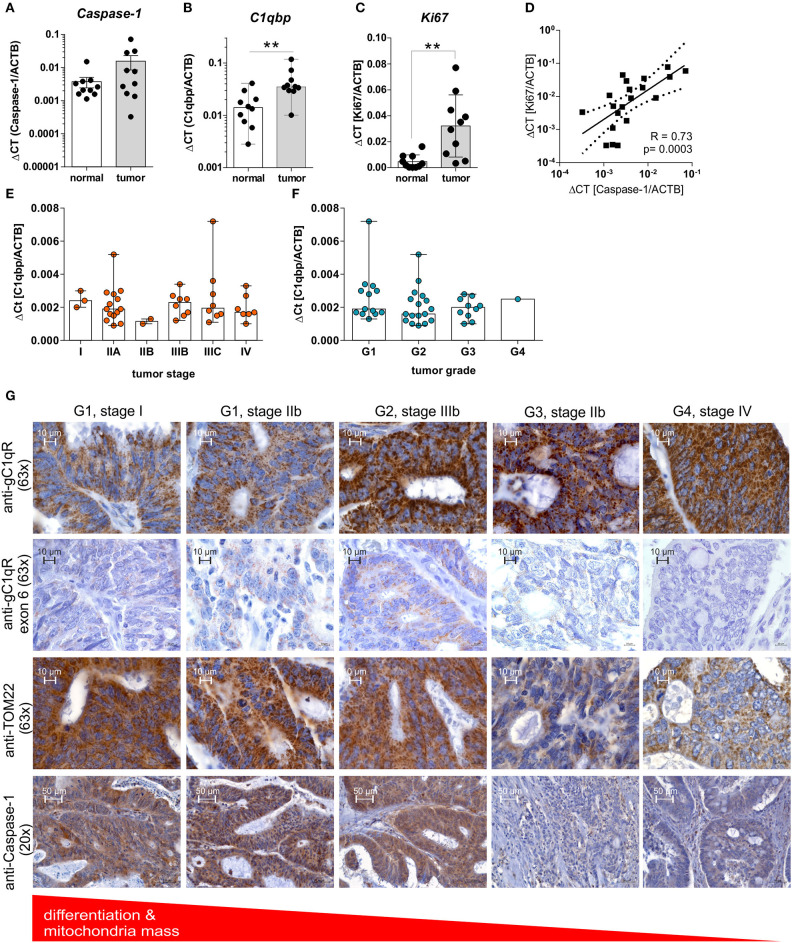
GC1qR expression is upregulated in CRC tissues and negatively correlates with the tumors' differentiation state. **(A–C)** QPCR analyses were performed to quantify mRNA expression level of **(A)** *Caspase-1*, **(B)** *C1qbp*, or **(C)** *Ki67* in 10 paired colonic normal or tumor tissues collected from CRC patients. **(D)** Results received from qPCR experiments for *Caspase-1* mRNA expression were put into relation with *Ki67* mRNA expression level. **(E,F)** *C1qbp* mRNA expression was quantified by qPCR experiments in a set of CRC tumor samples of different tumor **(E)** stage and **(F)** grade. **(G)** Immunohistochemistry analyses of tumor tissues displaying different grading states [grade 1 (G1), grade 2 (G2), grade 3 (G3), grade 4 (G4)] collected from CRC patients using primary antibodies specific for gC1qR (clone EPR8871; 63x magnification), gC1qR exon 6 (63x magnification), TOM22 (63x magnification) or Caspase-1 (20x magnification). **p ≤ 0.01.

Highly proliferating tumor cells are known to display an imbalance between glycolysis and OXPHOS activity, with a shift toward aerobic glycolysis, to ensure fast cell division (1). Based on our findings we demonstrated enhanced cell proliferation and a shift toward aerobic glycolysis after caspase-1 activation in gC1qR expressing cells (Figures 4, 5A–E) as well as decreased differentiation of colorectal carcinoma cells (Figures 5F–H), we hypothesized that grading and staging of colorectal carcinoma cells may be correlated with caspase-1 mediated cleavage of gC1qR. Therefore, we first analyzed mRNA expression of *Caspase-1, PYCARD*, and *NLRP3* in paired normal and tumor tissues collected from CRC patients (Table 1) by qPCR experiments. Unexpectedly, no differences between normal and tumor tissues were detected for analyzed transcripts (Figure 6A, Supplementary Figure 5A). Notably, significant up-regulation of *C1qbp* and the cell proliferation marker *Ki67* mRNA expression level were determined in tumor tissues compared to paired normal tissues (Figures 6B,C), while no correlation was found in colonic tissues between *C1qbp* and *Ki67* mRNA level, between *NLRP3* and *Ki67* mRNA level as well as between *PYCARD* and *Ki67* mRNA level (Supplementary Figures 5B–D). However, *Ki67* mRNA expression highly correlated with *Caspase-1* mRNA expression in colonic tissues, supporting our hypothesis that caspase-1 may be critically involved in the regulation of cell proliferation (Figure 6D). Furthermore, we tested *C1qbp* mRNA expression in 42 distinct CRC tumor samples of different tumor grades and stages. Unexpectedly, *C1qbp* mRNA expression was not affected by tumor stage or grade in analyzed CRC patient samples (Table 1, Figures 6E,F).

In the next set of experiments, protein expression level of gC1qR, TOM22, or full-length caspase-1 (Supplementary Figure 5E) were investigated by IHC experiments utilizing CRC tumor tissues of different grades (grade1-4) and stages (I-IV) (Table 1). Here, in contrast to healthy colonic tissue (Figure 1G, Supplementary Figure 1) protein expression of gC1qR negatively correlated with the mitochondrial marker TOM22 and was highest in grade 4 (G4) and lowest in grade 1 (G1) CRC (Figure 6G), although *C1qbp* mRNA expression did not correlate with tumor stage and grade (Figures 6E,F). Notably, staining of gC1qR-exon 6 and TOM22 protein correlated with inactive full-length caspase-1 staining (Figure 6G, Supplementary Figure 5G) in CRC samples with lowest expression level detected in high-grade CRC samples. These findings in combination with results from IHC experiments demonstrating gC1qR-exon 6 being detectable in all paired normal colon tissues from analyzed CRC patient samples (Supplementary Figure 5F) point to a mere post-translational processing of gC1qR potentially by active caspase-1, leading to the loss of mitochondrial biogenesis and an increase of non-mitochondria localized gC1qR protein level (Figure 6G, Supplementary Figure 5G).

## Discussion

Most tumor cells fine-tune their metabolism from balanced OXPHOS to fast but inefficient aerobic glycolysis, called the Warburg effect (3, 4). However, the question remains, whether regulation of the level of gC1qR localized in the mitochondria leads to a secondary regulation of energy provided by mitochondrial oxidative phosphorylation, thereby allowing the switch to aerobic glycolysis.

**Figure 7:**
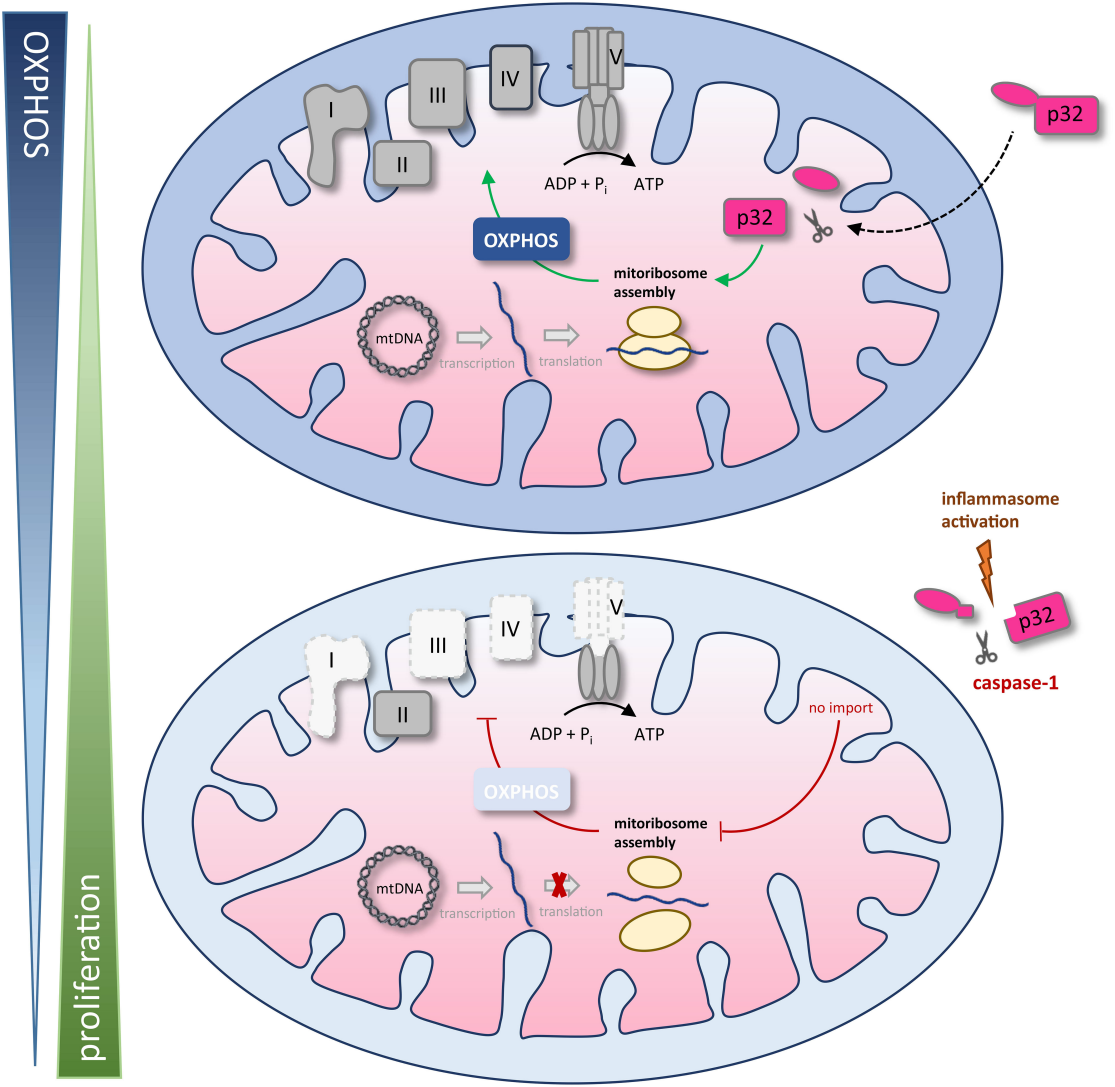
Schematic model of caspase-1 reduction of mitochondrial OXPHOS activity via gC1qR cleavage. The gC1qR protein encompasses an N-terminal mitochondria leader that mediates its import into the mitochondrial matrix. Recently, mitochondria located gC1qR has been demonstrated to be part of the mitoribosome and hence critically regulate translation of mitochondria encodes proteins such as complexes I, III, IV, and V of the respiratory chain. In the present study, two specific caspase-1 cleavage sites at asparagine residues 77 and 229 were identified in the amino acid sequence of gC1qR, resulting in the cleavage of its *N*-terminal mitochondrial leader under conditions that activate the inflammasome. As a consequence of gC1qR cleavage by active caspase-1, cells display a loss of OXPHOS activity and a shift toward aerobic glycolysis, enabling increased cell proliferation. mtDNA, mitochondrial DNA; OXPHOS, oxidative phosphorylation; I – V, complexes I to V.

We found that the amino acid sequence of gC1qR presents two specific caspase-1 cleavage sites at aspartic acid residues 77 and 229, resulting in the cleavage of the *N*-terminal mitochondrial leader. Furthermore, we functionally verified these cleavage sites by an *in vitro* cleavage assay followed by mass spectrometry based peptide sequencing. As a consequence of gC1qR cleavage by active caspase-1, tumor cells displayed a loss of OXPHOS activity and thereby an imbalanced OXPHOS and glycolysis activity. Excessive aerobic glycolysis activity then enabled augmented cell proliferation that was prevented by mutated caspase-1 cleavage sites (Figure 7).

The salient finding of the present study is that cleavage of gC1qR by active caspase-1 promotes aerobic glycolysis in tumor cells and boosts carcinogenesis. These data are in line with data from recent studies that demonstrated the NLRP3 inflammasome to be critical for tissue homeostasis in the colonic intestine by driving intestinal epithelial cell (IEC) proliferation and tissue repair under DSS-induced colitis conditions (30–32). Of note, mice deficient in caspase-1 displayed a hypoproliferative intestinal epithelium, while mice deficient in the intrinsic caspase-1 inhibitor, caspase-12, displayed exacerbated colitis-associated colorectal carcinogenesis due to increased IEC proliferation (30). These data reveal the NLRP3 inflammasome to drive IEC proliferation that is beneficial in the resolution of colitis but is detrimental in CRC. However, contradicting studies regarding the role of NLRP3 inflammasome activation in CRC development have been published ranging from tumor promoting (33) to tumor-preventing modes of action (34). Due to findings that the NLRP3 inflammasome is continuously activated by nutrient excess (9, 10), we propose a novel mechanism explaining how the exposome –for example Western diet–triggers self-sustained cell proliferation via caspase-1 mediated cleavage of gC1qR, thereby boosting fast cell proliferation under chronic inflammation and potentially inflammation-driven carcinogenesis.

In summary, the present study presents for the first time, an explanation on how the metabolic activity of gC1qR is controlled by the NLRP3 inflammasome and how this interplay impacts cellular balance between OXPHOS activity and glycolysis. This study therefore opens new alleys for novel strategies in the therapy of inflammation-driven carcinogenesis, including nutritional interventions that prevent activation of the inflammasome.

## Data Availability Statement

The original contributions presented in the study are publicly available. This data can be found here: FigShare (https://figshare.com/articles/dataset/gC1qR_peptide_sequencing_data_pdf/12886709).

## Ethics Statement

The studies involving human participants were reviewed and approved by ethical committee of the University of Lübeck. Written informed consent for participation was not required for this study in accordance with the national legislation and the institutional requirements.

## Author Contributions

SD designed the concept of the present study and supervised it. CS and SP collected and provided human biopsy samples. BG provided primary antibodies specific for distinct epitopes of human gC1qR. AS, AR, MH, HS, FF, A-KB, and SD performed the experiments and acquired the data. AS, AR, and SD analyzed and interpreted the data. AS, AR, and SD drafted the article. CS, CK, BG, and SD critically revised the article for important intellectual content. All authors read and approved the final manuscript.

## Conflict of Interest

BG receives royalties from the sale of monoclonal antibodies 60.11 and 74.5.2. The remaining authors declare that the research was conducted in the absence of any commercial or financial relationships that could be construed as a potential conflict of interest.
