# Supplementary material for: GC1qR Cleavage by Caspase-1 Drives Aerobic Glycolysis in Tumor Cells
Source: Front Oncol. 2020 Sep 30;10:575854. doi: 10.3389/fonc.2020.575854 (PMC7556196; doi:10.3389/fonc.2020.575854)
Supplement: Supplementary file 1 [file Data_Sheet_1.docx]

Supplementary Material

# Supplementary Figure 1

**
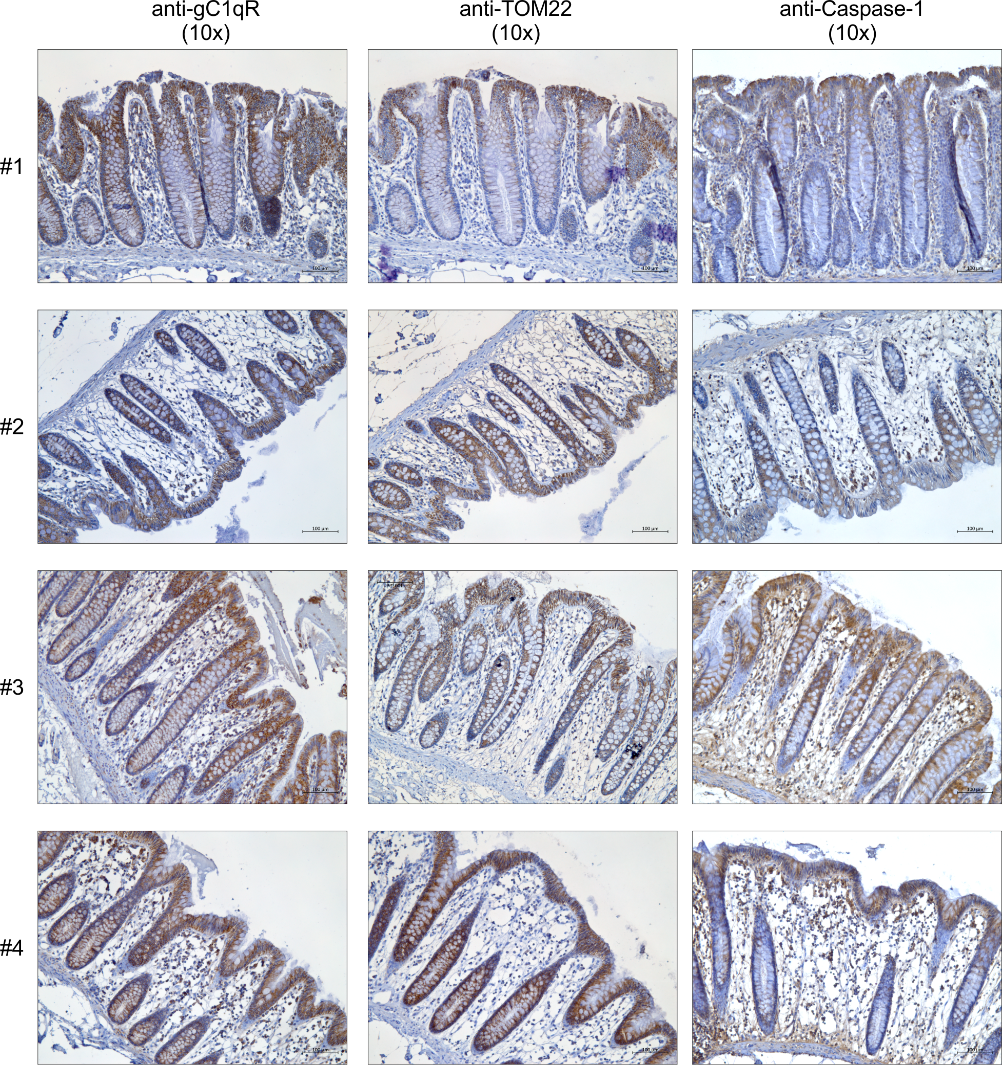
**

**Supplementary Figure 1.** Immunohistochemistry analyses of four independent paraffin-embedded formalin-fixed human colonic biopsy samples collected from normal tissue sites from CRC patients using anti-gC1qR Ab (clone EPR8871), anti-TOM22 Ab or anti-Caspase-1 Ab.

# Supplementary Figure 2


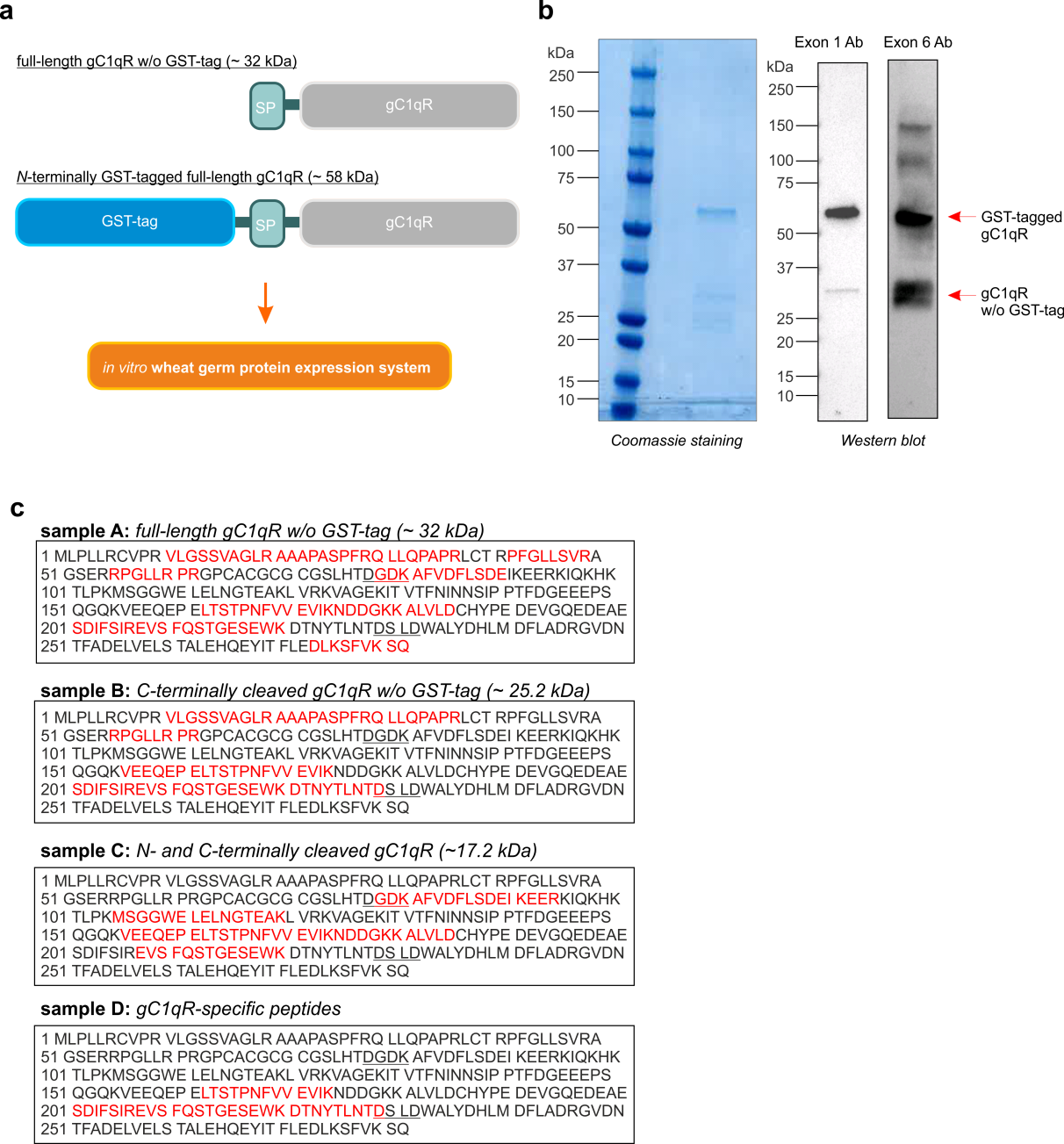


**Supplementary Figure 2.** (a) Schematic model of utilized *N*-terminally GST-tagged human gC1qR protein that has been generated using an *in vitro* wheat germ protein expression system. (b) Human recombinant gC1qR protein was separated under reducing conditions by SDS-PAGE and proteins were visualized by coomassie blue staining (left panel) or by Western blot experiments using distinct gC1qR-directed primary antibodies with different binding epitopes located in exon 1 or exon 6. (c) *In vitro* cleavage assay was performed by incubating human recombinant gC1qR in the presence of human recombinant active caspase-1 (~10 and 20 kDa) at 37°C for 24 hours. Afterwards, reduced protein samples were separated by SDS-PAGE and proteins were visualized by Coomassie blue staining. Four protein bands were cut out of the gel. Protein spots were in-gel digested by trypsin and analyzed by nanoHPLC-ESI-MS/MS method. Detected sequences in according bands are marked in red, predicted caspase-1 cleavage sites are underlined.

# Supplementary Figure 3

**
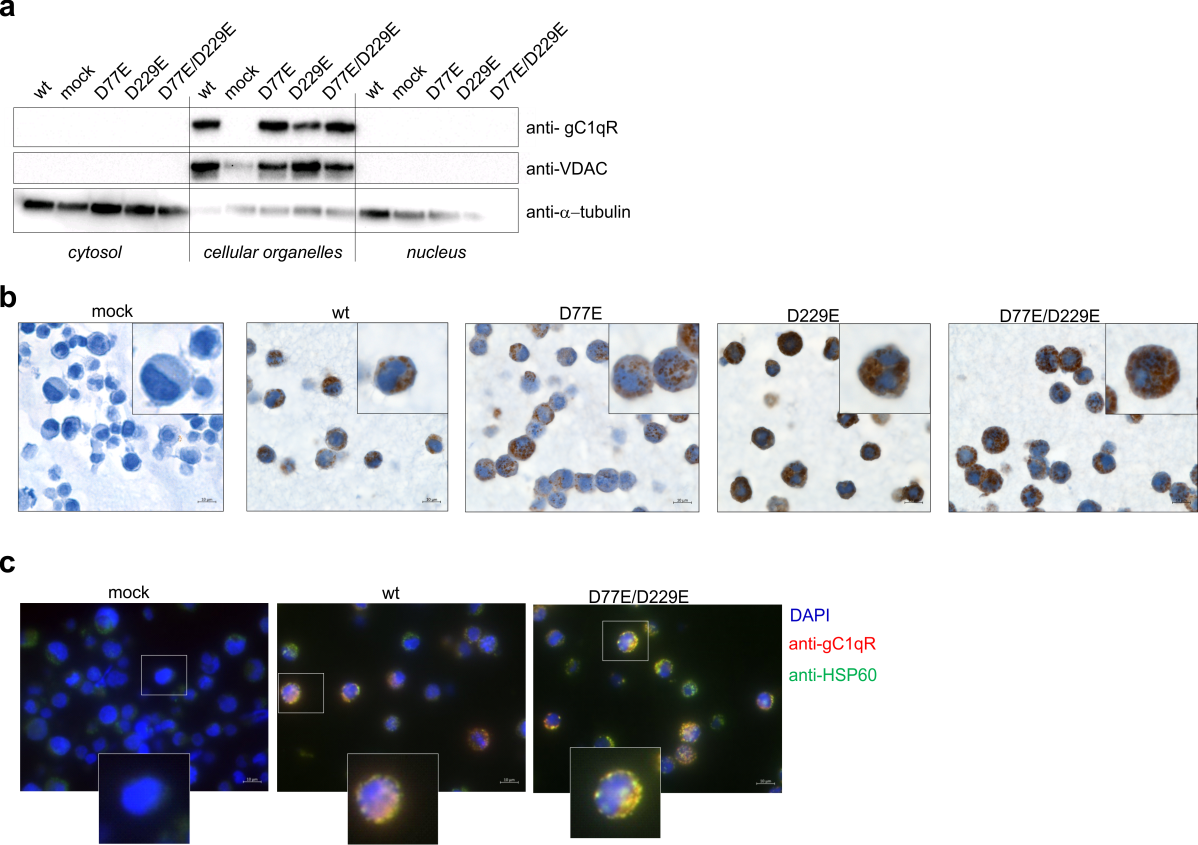
**

**Supplementary Figure 3.** (a) Site-directed mutagenesis of human *C1qbp* was performed using site-specific oligonucleotides to prevent caspase-1 cleavage of gC1qR. Aspartic acid (D) residues at aa77 and aa229 were substituted by glutamic acid (E) residues, resulting in the following gC1qR mutants D77E, D229E or D77E/D229E. HAP1-gC1qR^-/-^ cells were stably transfected with generated plasmids encoding wt or mutated *C1qbp* variants. Reduced protein samples isolated from the cytosolic fraction, the cellular organelle fraction (including mitochondria) or from the nuclear fraction from HAP1-gC1qR transfectants w/o NAC were separated by SDS-PAGE. Western blot experiments were performed using the anti-gC1qR antibody clone EPR8871, an anti-VDAC antibody or an anti-α-tubulin antibody. (b) HAP1-gC1qR mutant cell lines were analyzed by immunohistochemistry experiments using a gC1qR-specific antibody specific for epitopes located in exon 3. (c) Co-localization of gC1qR-wt or gC1qR-D77E/D229E with HSP60 protein was assessed by fluorescence microscopy.

# Supplementary Figure 4

**
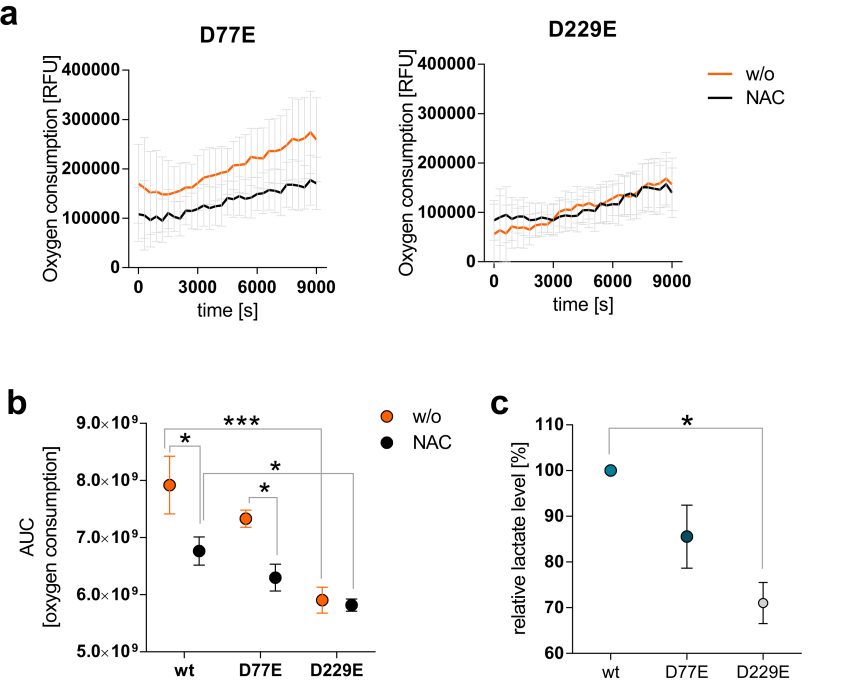
**

**Supplementary Figure 4.** (a) Time-dependent measurement of oxygen consumption rate of HAP1 transfectants in the presence or absence of over-expressed inflammasome components (NAC). (b) The area under the curve of data presented in (a) was calculated for each single experiment and each cell line. (c) Lactate production was measured in cell culture supernatants after 72 hours of incubation of HAP1-gC1qR wt or indicated mutant cell lines stably transfected with NAC. Lactate levels generated by gC1qR-mutants were related to lactate levels produced by gC1qR-wt cells. Results are expressed as mean ± SEM and are presented from at least three independent experiments. * p≤0.05, *** p≤0.001, **** p≤0.0001.

# Supplementary Figure 5

**
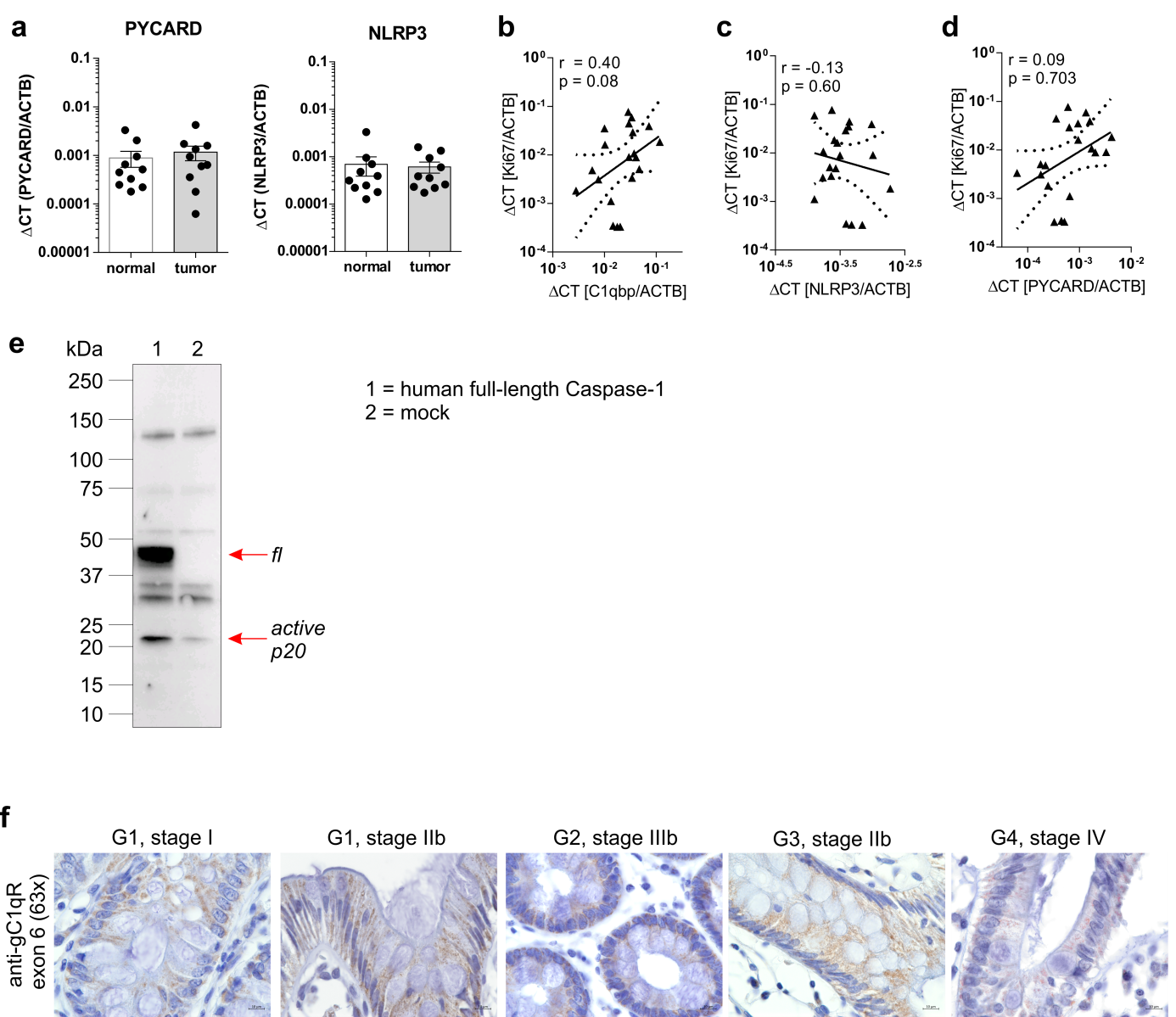
**

**g**

**
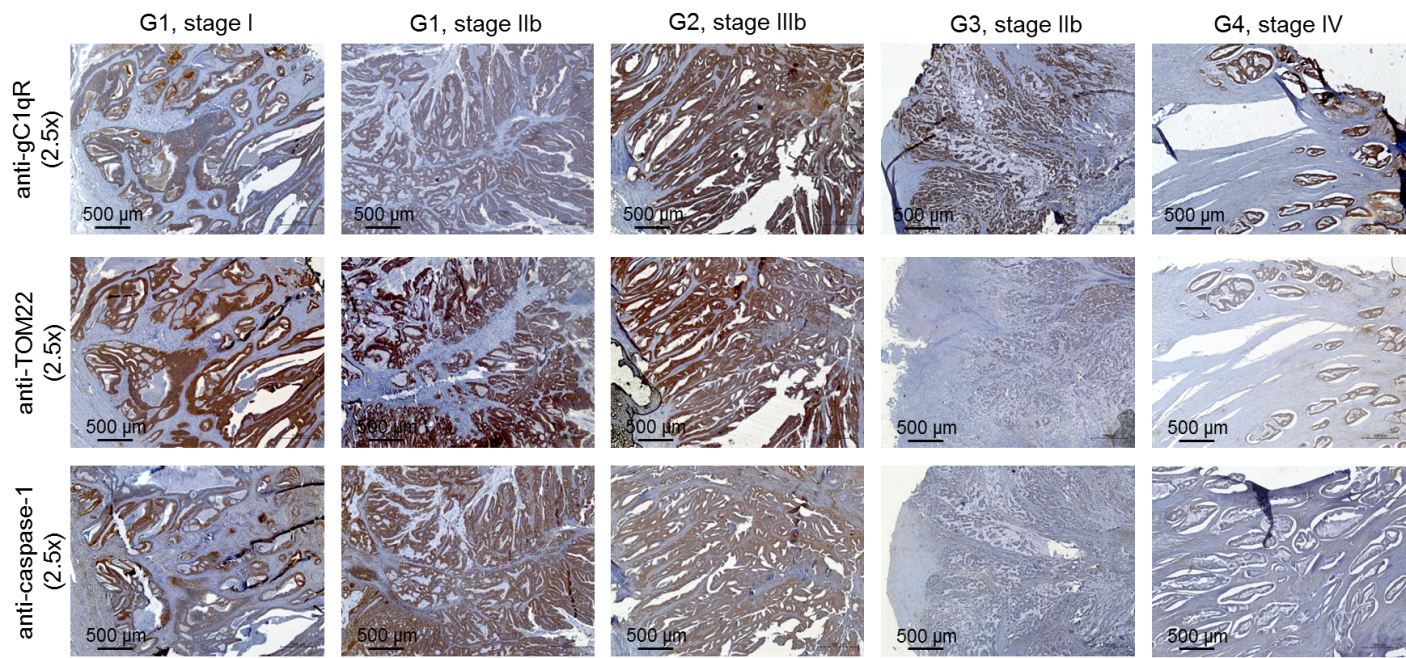
**

**Supplementary Figure 5.** (a-d) QPCR analyses were performed to quantify mRNA expression level of *PYCARD*, *NLRP3*, *C1qbp* or *Ki67* in ten paired colonic normal or tumor tissues collected from CRC patients. Results received from qPCR experiments for *Ki67* mRNA expression were put into relation with (b) *C1qbp* mRNA expression level, with (c) *NLRP3* mRNA expression level or with (d) *PYCARD* mRNA expression level. (e) Reduced whole protein fractions isolated from HAP1-gC1qR-wt cells transfected with a plasmid encoding full-length human Caspase-1 or with a mock plasmid were separated by SDS-PAGE. Western blot experiments were performed using a primary antibody specific for human Caspase-1. fl = full-length, p20 = cleavage fragment p20 of active caspase-1. (f) Immunohistochemistry analyses of five independent paraffin-embedded formalin-fixed human colonic biopsy samples collected from normal tissue sites from CRC patients using an anti-gC1qR exon 6 Ab. (g) Immunohistochemistry analyses of tumor tissues displaying different grading states (grade 1 (G1), grade 2 (G2), grade 3 (G3), grade 4 (G4)) collected from CRC patients using primary antibodies specific for gC1qR (clone EPR8871; 2.5x magnification), TOM22 (2.5x magnification) or Caspase-1 (2.5x magnification).
